# Application of Glycation in Regulating the Heat-Induced Nanoparticles of Egg White Protein

**DOI:** 10.3390/nano8110943

**Published:** 2018-11-15

**Authors:** Chenying Wang, Xidong Ren, Yujie Su, Yanjun Yang

**Affiliations:** 1 State Key Laboratory of Food Science and Technology and School of Food Science and Technology, Jiangnan University, Wuxi 214122, China; chenying071776@163.com; 2 State Key Laboratory of Biobased Material and Green Papermaking, Qilu University of Technology, Shandong Academy of Sciences, Jinan 250353, China; renxidong1986@126.com; 3 Shandong Provincial Key Laboratory of Microbial Engineering, Department of Bioengineering, Qilu University of Technology, Shandong Academy of Sciences, Jinan 250353, China

**Keywords:** egg white protein, isomalto-oligosaccharide, glycation, thermal aggregation, nanoparticle, emulsifying property

## Abstract

Due to the poor thermal stability of egg white protein (EWP), important challenges remain regarding preparation of nanoparticles for EWP above the denaturation temperature at neutral conditions. In this study, nanoparticles were fabricated from conjugates of EWP and isomalto-oligosaccharide (IMO) after heating at 90 °C for 30 min. Meanwhile, the effects of protein concentration, temperature, pH, ionic strength and degree of glycation (DG) on the formation of nanoparticles from IMO-EWP were investigated. To further reveal the formation mechanism of the nanoparticles, structures, thermal denaturation properties and surface properties were compared between EWP and IMO-EWP conjugates. Furthermore, the emulsifying activity index (EAI) and the emulsifying stability index (ESI) of nanoparticles were determined. The results indicated that glycation enhanced thermal stability and net surface charge of EWP due to changes in the EWP structure. The thermal aggregation of EWP was inhibited significantly by glycation, and enhanced with a higher degree of glycation. Meanwhile, the nanoparticles (<200 nm in size) were obtained at pH 3.0, 7.0 and 9.0 in the presence of NaCl. The increased thermal stability and surface net negative charge after glycation contributed to the inhibition. The EAI and ESI of nanoparticles were increased nearly 3-fold and 2-fold respectively, as compared to unheated EWP.

## 1. Introduction

Egg white protein (EWP) is an important ingredient in food processing, because of its abundant nutritive value and various functional properties. The main components of EWP are ovalbumin (OVA, 54%), ovotransferrin (OT, 12%), ovomucoid (OM, 11%) and lysozyme (LY, 3.4%) [1]. These proteins mainly show a globular structure. However, its industrial application is limited by its poor thermal stability and emulsifying properties. It was reported that heat-induced nanoparticles formed from globular proteins could increase their emulsifying capacity and binding ability to hydrophobic bioactive compounds [2,3,4]. Although half of the amino acid residues in OVA are hydrophobic, EWP presents good water-solubility, since most hydrophobic amino acid residues are embedded into protein molecules under natural conditions. Heating promotes EWP unfolding, in which hydrophobic amino acids are exposed, conferring an increase in protein surface hydrophobicity [5]. However, considering the thermolability of EWP, and in particular the OT (denaturation temperature *TD* = 62 °C, p*I* = 6.1) [6], the aggregation of which occurs easily under neutral conditions, the application of thermal modification technology in improving the functional properties of EWP is limited. Attempts in fabrication of EWP nanoparticles by heat treatment under extremely acidic [7] or alkaline conditions [8] have been successful. This proves that the heat-induced EWP nanoparticles exhibit excellent potential to be a type of delivery system for hydrophobic compounds. However, the application systems for extremely acidic or alkaline conditions are limited. Therefore, the preparation of nanoparticles suitable for neutral systems is very important. However, the stable heat-induced nanoparticle prepared by commercial EWP at neutral condition was not reported.

Glycation by Maillard reaction (MR) between reducing sugar and free amino group in proteins to form cross-links are one of the few chemical modification methods applicable to food production. Many studies have succeeded in suppressing thermal aggregation of proteins using glycation, such as whey protein [9], soy protein [10], peanut protein [11] and superoxide dismutase [12]. In addition, the MR would partially unfold the EWP molecules and expose the hydrophobic group to the surface, further improving the oil-in-water emulsifying ability [13]. Therefore, preparation of heat-induced nanoparticles from glycated EWP may be more beneficial for the emulsion system.

In this study, the isomalto-oligosaccharide (IMO) and EWP conjugates (IMO-EWP) were prepared by dry-heating MR. Sodium dodecyl sulfate-polyacrylamide gel electrophoresis (SDS-PAGE) and Fourier transform infrared spectroscopy (FTIR) were first used to characterize the structural changes of EWP after glycation. Then, a comparative study of thermal denaturation properties, surface hydrophobicity and ζ-potential were conducted at neutral conditions. The effects of temperature, pH and ionic strength on the thermal aggregation of IMO-EWP dispersions were further investigated. Finally, emulsifying properties were examined to verify the functional properties of fabricated nanoparticles. This study is expected to provide useful information for the preparation of EWP nanoparticles and expand the application of EWP in emulsion systems.

## 2. Materials and Methods 

### 2.1. Materials

Hen EWP powder was provided by Rongda Co., Ltd. (Xuancheng, China). The EWP powder was manufactured from fresh egg white after removing glucose, followed by spray-drying Isomalto-oligosaccharide-900 (IMO) with an average molecular weight of 564 Da and reducing sugar content of 21.52% was purchased from Baiyou Bio-Technology Co., Ltd. (Langfang, China). The composition of IMO (Figure S1 and Table S1) is shown in Supplementary Materials. Other reagents were purchased from Sinopharm Chemical Reagent Co., Ltd. (Shanghai, China).

### 2.2. Preparation of IMO-EWP Conjugates

IMO and EWP were separately mixed together at weight ratios of 1:2, 1:10 and 1:40 (the IMO ratios were also indicated as the weight ratio of IMO to protein of 2.5%, 10% and 50%) and dissolved in distilled water, then adjusted to pH 7.0 with 0.1 M NaOH and 0.1 M HCl. After centrifuging (6000× *g*, 30 min) and filtering, the supernatant was collected and freeze-dried. The freeze-dried EWP without IMO addition was prepared the same as the mixtures. The resulting freeze-dried EWP and mixtures were then placed at 60 °C for 3 days at a relative humidity of 79%. As a control, EWP and IMO were individually maintained for 3 days under the same conditions. The samples were dissolved and dialyzed (molecular mass cut off 3.5 kDa) against distilled water for 3 days at 4 °C to remove the unreacted IMOs. After freeze-drying, the samples were stored at 4°C before further experimentation. The heated conjugates were designated as 2.5% IMO-EWP, 10% IMO-EWP and 50% IMO-EWP, respectively. The protein content of freeze-dried EWP and conjugates were measured via Kjeldahl method.

### 2.3. Sodium Dodecyl Sulfate-Polyacrylamide Gel Electrophoresis (SDS-PAGE)

Reducing SDS-PAGE was performed on slab gels (12% separating gel and 5% stacking gel) [13]. Samples were dissolved or diluted to a protein concentration of 2.5 mg/mL. The sample supernatants after centrifugation were added into each lane after mixing with sample loading buffer. 0.2% Coomassie Brilliant Blue G-250 and 0.5% periodic acid fuchsin (PAS) were used to stain the protein and carbohydrates in the gel, respectively. Bovine albumin was used as negative control for PSA glycoprotein staining test.

### 2.4. Grafting Degree (DG) of the IMO-EWP Conjugates

*O*-phthalaldehyde (OPA) method [14] was employed to measure the free amino groups of proteins. Two hundred microlitres of sample were mixed with 4 mL of OPA solution. Distilled water was used as blank. The absorbances were measured at 340 nm.

The DG of native EWP and IMO-EWP conjugates were calculated as follows [13]:

DG% = (*A*_0_ − *A*_c_)/*A*_0_ (1)

where *A*_0_ and *A*_c_ are free amino groups content of native EWP and conjugates, respectively.

### 2.5. Fourier Transform Infrared Spectroscopy (FTIR)

The secondary structural changes of conjugates were analyzed by FTIR. An FTIR IS10 spectrometer (Nicolet Co., Madison, WI, USA) was used to determine the FTIR spectra of native and glycated EWPs that were previously deposited on infrared-transparent sodium bromide (KBr) windows. The FTIR spectra were measured from 4000 to 400 cm^−1^ for 16 scans. After being baseline corrected, and the area was normalized between 1600 and 1710 cm^−1^ using the PeakFit v4.12 (SeaSolve, Framingham, MA, USA). Quantitative estimation of secondary structure components was performed using Gaussian peaks and curve-fitting models [15].

### 2.6. Differential Scanning Calorimetry (DSC)

The thermal property was measured on a TA Q2000-DSC thermal analyzer (TA Instruments, New Castle, PA, USA) according to the method previously described by Liu [11] with some modification. Samples (protein concentration of 35%, *w*/*v*; pH 7.0) were heated from 30–100 °C at a linear rate of 5 °C /min. TA Universal Analysis 2000 was used to calculate enthalpy changes of denaturation (Δ*H*) and the denaturation temperature (*T*_d_).

### 2.7. Measurement of Surface Hydrophobicity (H_o_)

Samples were dissolved in distilled water to a soluble protein concentration of 10 mg/mL. Then the filtered protein solutions were diluted with 10 mM phosphate buffer (pH 7.0) to a series of five protein concentrations ranging from 0.005–0.02% *w*/*w*. The surface hydrophobicity of treated samples was determined using the fluorescence probe of 1-anilino-8-naphthalenesulfonate (ANS) [16]. The fluorescence intensity (FI) was measured with an F-7000 spectrofluorometer (Hitachi, Tokyo, Japan). The initial slope of the FI-protein concentration (mg/mL) plots was used as an index of *H*_o_.

### 2.8. Preparation of Heat-Induced Aggregate Particles

EWP and IMO-EWP conjugates were separately dissolved in distilled water to corresponding soluble protein concentrations (1–5%, *w*/*v*). Then the solutions were adjusted to pH 3.0–9.0 with 0.5 M NaOH and HCl, and 0–150 mM NaCl was added. All protein solutions were filtered before heating. Five-milliliter aliquots of protein dispersions were added to a capped glass vial (10 mL). For the temperature test, samples were incubated at 60–90 °C for 30 min; for the test of protein concentration, pH and ionic strength, samples were incubated at 90 °C for 30 min in a water bath. After heating, the vials were immediately transferred to an ice-water bath, in which the heat-induced particle dispersions were maintained at 4 °C.

### 2.9. Turbidity Measurement

Particle dispersions (1%, *w*/*v*) were diluted with distilled water at a ratio of 1:10. The absorbances of sample solutions at 500 nm were measured with a UH5300 spectrophotometer (Hitachi, Tokyo, Japan). Turbidity was expressed by light transmittance.

### 2.10. Determination of Z-Average Hydrodynamic Diameters and ζ-Potentials

A Zetasizer Nano ZS instrument (Malvern Instruments, Worcestershire, UK) was employed to measure diameters of particles and the ζ-potential [17]. For Z-average hydrodynamic diameter measurement, the protein dispersion/emulsion was diluted with double-distilled water (1:200, *v*/*v*). After equilibrating for 120 s, samples were measured with 15 sequential readings. For ζ-potential measurement, the protein dispersions/ emulsions were diluted at a ratio of 1:200 (*v*/*v*) using double-distilled water with equivalent pHs. Samples were measured in triplicate, 12 sequential readings per measurement.

### 2.11. Atomic Force Microscope (AFM)

The surface morphology of particles was measured using AFM [7]. The protein solutions were diluted to a protein concentration of 5 μg/mL. A droplet (1–3 μL) of prepared sample was spread on a freshly cleaved mica disk and air dried naturally at room temperature. The images were obtained using a Dimension ICON microscope (Bruker Corporation, Jena, Germany).

### 2.12. Emulsifying Activity Indexe (EAI) and Emulsifying Stability Index (ESI)

EAI and ESI were determined by turbidimetric method [18]. Samples were dissolved or diluted with distilled water and adjusted to pH 7.0 with 0.1 M NaOH and HCl to obtain 0.1% (*w*/*v*) aqueous solutions. 10 mL of corn oil in 30 mL of sample solution was emulsified by an Ultra-Turrax blender (IKA T25 Basic, Staufen, Germany) at 11,000 rpm for one minute at room temperature. One hundred microlitres of the emulsions were pipetted from the bottom of the tube into 5 mL of SDS solutions (0.1%, *w*/*v*) immediately (0 min) and 10 min after homogenization. The turbidity of the diluted solutions was then determined at 500 nm. The EAI was the absorbance taken immediately after emulsification. The ESI values were calculated using the following equations:

(2) $$\mathrm{ESI}\;(\min)\;=\;A_{0}\;\times \;10/(A_{0}\;-\;A_{10})$$

where *A*_0_ and *A*_10_ are the absorbances of diluted emulsions at 0 and 10 min, respectively.

### 2.13. Statistical Analysis

To check reproducibility, tests were carried out in triplicate. Results were expressed as the mean value ± standard deviation (SD) of three independent treatments. Analysis of variance was used to calculate the significance of the samples, and sample means were separated using the Student’s paired *t*-test. Differences were considered significant at *p* < 0.05.

## 3. Results and Discussion

### 3.1. Structure Characteristics of IMO-EWP Conjugates

**Figure 1:**
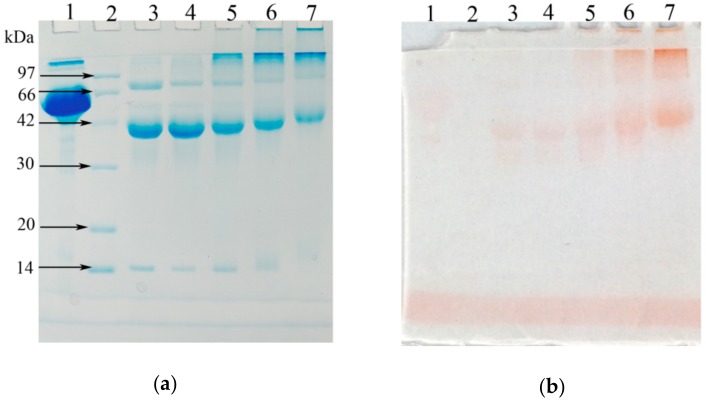
SDS-PAGE profiles of egg white protein (EWP) and isomalto-oligosaccharide (IMO)-EWP conjugates stained for proteins (**a**) or saccharides (**b**). Lane 1: Bovine albumin (negative control); Lane 2: marker proteins; Lane 3: EWP; Lanes 4: EWP incubated at glycation condition for 3 days; Lane 5–7: IMO-EWP conjugates with sugar-protein weight ratios of 2.5%, 10% and 50%, respectively.

The degree of glycation (DG) of 2.5%, 10% and 50% IMO-EWP were 62.73, 68.17 and 71.05, respectively, which indicated that the higher IMO ratios resulted in higher DG of glycated EWP. Electrophoretic patterns of EWP and its conjugates with IMO were shown in Figure 1. Native EWP was mainly composed of three major bands approximately at 14, 45 and 76 kDa, which correspond to lysozyme (LZ), ovalbumin (OVA) and ovotransferrin (OT), respectively [19]. After the MR, changes happened in the MW as seen by the densities of the bands (lanes 3–5). The bands of LZ, OVA and OT shifted to higher MW, and a large number of continuous bands appeared on the top of the conjugate electrophoretic patterns, indicating the formation of protein polymers in glycated EWP. The results were consistent with previous studies [13,20]. The MW (Figure 1a) and saccharide moieties (Figure 1b) of LZ, OVA and OT gradually increased with the increase of DG, which further confirmed the formation of conjugates (Table 1).

**Figure 2:**
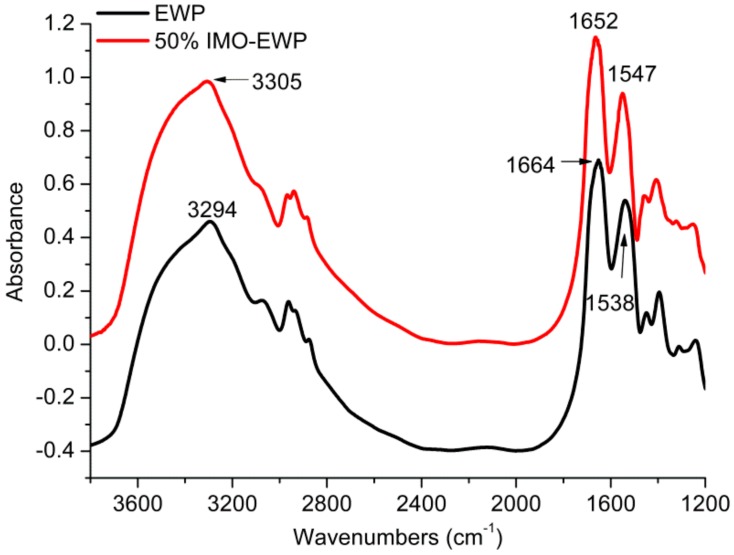
Fourier transform infrared spectroscopy (FTIR) spectra of EWP and 50% IMO-EWP conjugate at pH 7.0.

FTIR was employed to analyze the secondary structure of EWP and 50% IMO-EWP conjugate (Figure 2). The hydroxyl stretching bands (~3300 cm^−1^) of IMO-EWP sample showed stronger absorption, indicating that the conjugation of IMO could increase the hydroxyl content of EWP. Meanwhile, the absorption peaks of EWP at 1652 and 1538 cm^−1^ shifted to 1664 and 1547 cm^−1^ after glycation. A previous study reported that the amide I absorption band at 1664 cm^−1^ represent the structure formation of Schiff bases with spectral overlap of the C=O group, coupled with in-plane –NH bending and C=N linkage [21], and the absorption band at 1538 cm^−1^ was attributed to the primary amino group [22]. The above results suggested that the condensation reaction of primary amino groups of protein with carbonyl groups of reducing sugar was happened to form Schiff base products with the release of water and consumption of amino groups.

### 3.2. Thermal Denaturation Properties of IMO-EWP Conjugates 

**Figure 3:**
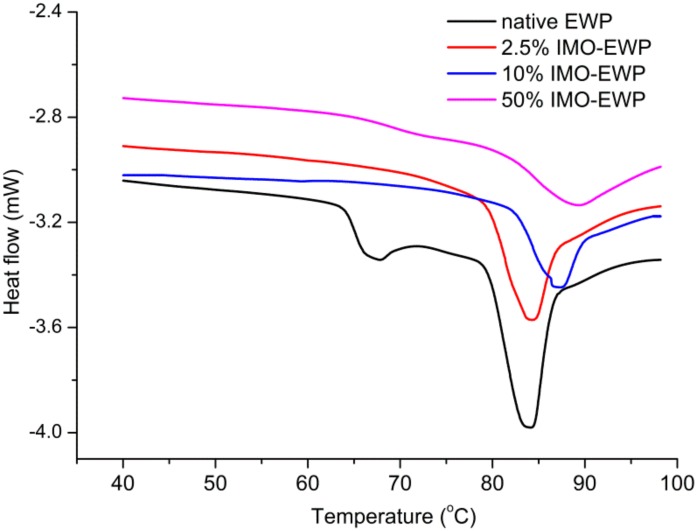
Differential Scanning Calorimetry (DSC) thermograms of EWP and IMO-EWP conjugates at pH 7.0.

The DSC curves of EWP and IMO-EWP conjugates during programmed heating at pH 7.0 are shown in Figure 3. The EWP exhibited two major endothermic transitions: the first peak at 66.56 ± 0.03 °C, which mainly raised from the denaturation of OT, and the second peak at 84.11 ± 0.05 °C indicated the denaturation of OVA. These results are similar to previous studies [23]. Notably, the endothermic peak of OT in IMO-EWP conjugates disappeared and the endothermic peak of OVA broadened with an increase of IMO ratio during glycation. It is reported that the broadening of the peak indicates the existence of denatured intermediates different from the native form [24]. This intermediate state, referred to as ‘molten globule’ state [25], maintained a native like secondary structure but tends to lose some of its tertiary structure. Because the temperature of MR was near the denaturation temperatures (*T*_d_) of OT, the OT turned to ‘molten globule’ during glycation. In addition, glycation partially denatured the OVA to ‘molten globule’ state and increasing IMO ratios could significantly promote the conformation changes. The DSC characteristic changes for heat denaturation of EWP and IMO-EWP conjugates are summarized in Table 1. The *T*_d_ of OVA in the conjugates was significantly higher than that of EWP, and *T*_d_ which elevated with an increase in IMO ratios. Generally, for a globular protein, a higher *T*_d_ is related to higher thermal stability [11]. Thus, the thermal stability of EWP was remarkably improved by glycation, in agreement with previous studies [11,13]. Conversely, the enthalpy changes (Δ*H*) of OVA in the conjugates were lower, as compared to EWP. With an increase of IMO ratio, the Δ*H* value of OVA gradually decreased. The lower Δ*H* values of OVA in the conjugates were attributed to the partial unfolding of OVA during glycation [11]. In conclusion, glycation could change the conformation of EWP and significantly improve its thermal stability.

### 3.3. Surface Hydrophobicity (H_o_) and ζ-Potential of IMO-EWP Conjugates

Hydrophobic interaction is a major attractive intermolecular force to facilitate protein aggregation, and therefore, the *H*_o_ of proteins was measured using the ANS method. The *H*_o_ of 2.5%, 10% and 50% IMO-EWP were 8742 ± 236, 6074 ± 101 and 3102 ± 1391, respectively, while that of EWP was 1313 ± 955. The increased *H*_o_ of the conjugates was much higher than that of the EWP. Previous studies suggest that denaturation of OVA during glycation would unfold the native tertiary structure and expose the buried hydrophobic groups [13,24]. The changes in structure of EWP may relate to the decrease of Δ*H* values and the improvement of thermal stability after glycation (Table 1). In addition, the *H*_o_ is highest for 2.5% IMO-EWP and gradually decreased with the increase of DG, which was due to that the increased attachment of sugar chains decreasing the hydrophobicity of proteins [26].

**Figure 4:**
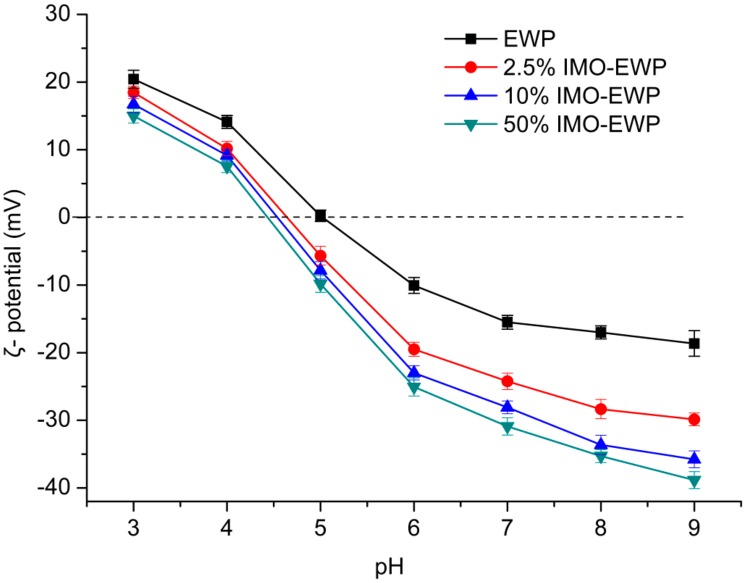
ζ-potentials of EWP and IMO-EWP conjugates at pH 3.0–9.0.

As shown in Figure 4, the ζ-potentials of conjugates were consistently lower compared to EWP. When pH was above p*I*, the absolute values of ζ-potentials for the conjugates were much higher than EWP, which means more electrostatic repulsion among the IMO-EWP molecules. It has been reported that the reducing end carbonyl groups of saccharides were mainly attached to the lysine and arginine residues (positively charged) of proteins during glycation, which would reduce the positive electrical charges [27]. On the other hand, more charged amino acids would be exposed to the exterior due to the protein unfolding caused by the dry-heating process of glycation.

### 3.4. Effects of Temperature, Protein Concentration, pH and Ionic Strength on the Formation of Nanoparticles

**Figure 5:**
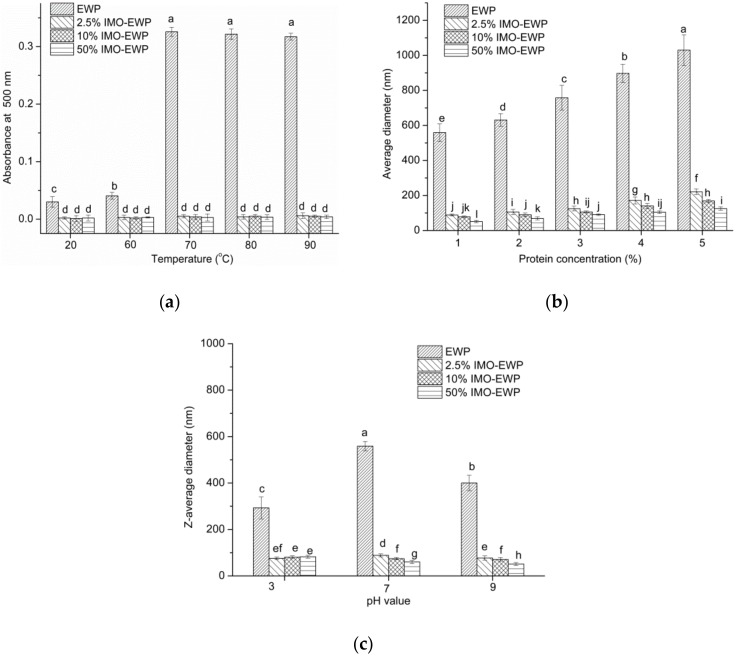
Turbidity of the heat-induced particles formed from EWP and IMO-EWP conjugates at different temperatures (**a**). Z-average hydrodynamic diameters of heat-induced particles formed at different protein concentrations (**b**) and pHs (**c**) from EWPs and conjugates. Different letters (a–l) indicate significant differences (*p* < 0.05).

Heating temperature has a significant influence on particle size. Changes in turbidity could reflect the sizes of the heat-induced particles. As shown in Figure 5a, the turbidity of the heat-induced particles formed from the EWP were significantly increased when the heating temperatures were above 60 °C, whereas the heat-induced particles formed from the IMO-EWP conjugates scarcely changed their turbidity and remained transparent with the increase of heating temperatures. Because the hydrophobic interactions were strengthened after glycation (Table 1), the inhibition of conjugate aggregation after heating may be attributed to the increased thermal stability of OT (Figure 3) and electrostatic repulsion (Figure 4). Sponton et al. also found that the electrostatic repulsions among EWP anionic charges at extremely alkaline pH were the dominating mechanism responsible for the suppression of thermal aggregation [8]. 

**Figure 6:**
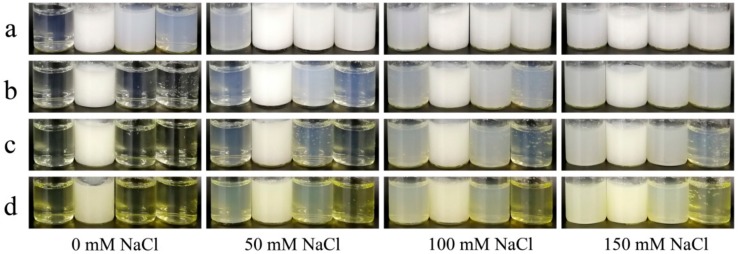
Photographs of EWP and IMO-EWP heat-induced particles prepared at different pHs and NaCl concentrations. EWP (**a**), 2.5% IMO-EWP (**b**), 10% IMO-EWP (**c**) and 50% IMO-EWP (**d**). Samples were adjusted to pH 3.0, 5.0, 7.0, 9.0 (vials from left to right in each image) and 0–150 mM NaCl was added as appropriate before heating.

**Figure 7:**
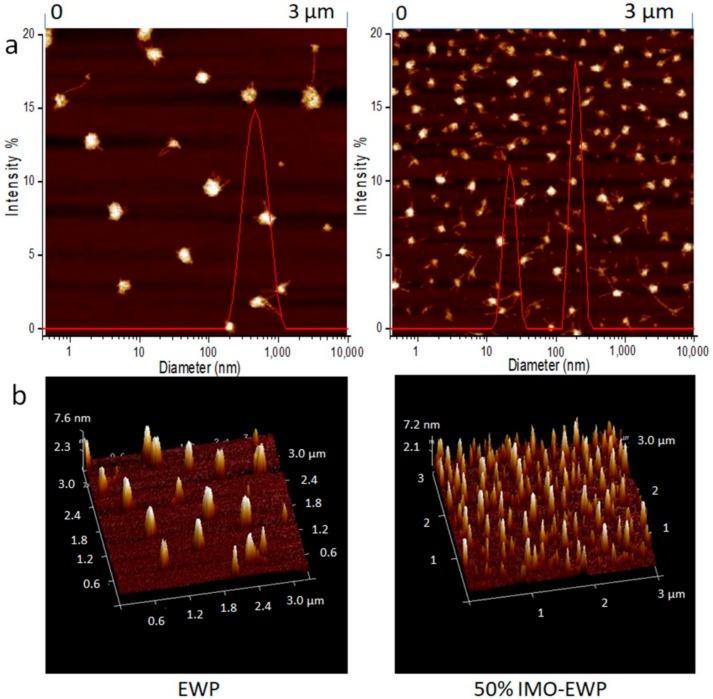
Atomic force microscope (AFM) images and particle diameter distributions of heat-induced particles from EWP and 50% IMO-EWP conjugate. 2D images and particle diameter distributions (**a**); 3D images (**b**).

As shown in Figure 5b, the particle size of aggregates increased with protein concentration. This could be explained by a rise in the number of protein macromolecules increasing the molecular collision, promoting heat-induced aggregation [28]. Besides, the diameters of the particles produced from conjugates were <200 nm, and considered “nanoparticles”. The effects of pH and ionic strength on the formation of heat-induced particles were also considered (Figure 5c and Figure 6). The particle dispersions of the conjugates were yellowish and the colors were deepened with increased of DG, which was a characteristic of nonenzymatic browning due to MR. It can be seen that the overall particle dispersions showed turbid appearance (Figure 6) and large particle size at pH 5.0 (the sizes of EWP aggregates at pH 5.0 were too large to be determined by DLS), which would indicate the presence of protein aggregates [1]. This was a result of the electrostatic repulsion being weakened as pH approaches p*I* [29]. Without the addition of NaCl, the heat-induced particles formed from the IMO-EWP conjugates maintained their clarity at pH 3.0, 7.0, and 9.0, while the particles from the EWP samples were transparent only at pH 3.0. This was consistent with the diameter results (Figure 5c). The increased electrostatic repulsion induced by glycation was responsible for the transparency of nanoparticles at pH 7.0–9.0. However, the transparent particle dispersions became turbid when NaCl was added, and the turbidity became more severe with the increased addition of NaCl. This was due to the reduction in electrostatic repulsion among the protein molecules caused by binding or shielding of NaCl [30]. Besides, the particles formed from the conjugates with higher DG showed more clarity appearance at the same pH and NaCl conditions (Figure 7c,d). These results again agreed with the decreased particle size with the increase of DG (Figure 5c). In conclusion, the heat-induced particles tended to lower sizes with the increase of DG, pH and ionic strength at lower protein concentrations.

To confirm the micromorphology properties, the heat-induced particles fabricated by EWP and conjugate at pH 7.0 were observed under AFM (Figure 7). The dimension of the conjugate particle was much smaller than EWP particle, which agreed with the hydrodynamic diameter determined by DLS (Figure 5).

### 3.5. Emulsifying Activity Index (EAI) and Emulsifying Stability Index (ESI) of Nanoparticles

**Figure 8:**
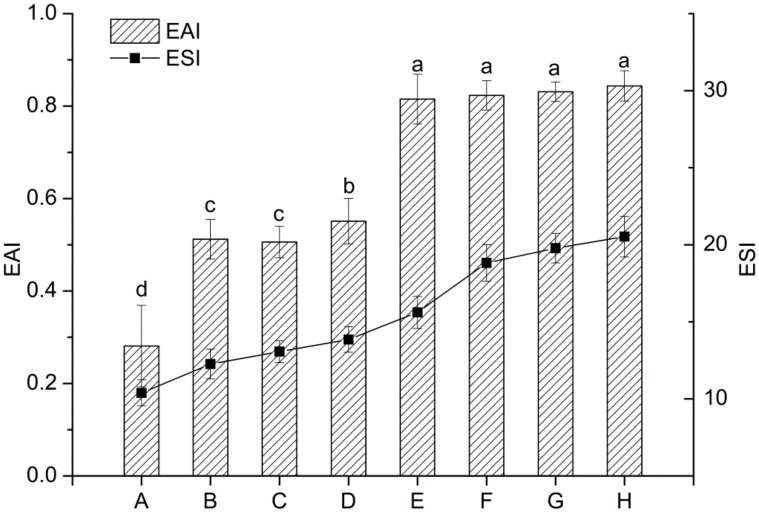
Emulsifying activity and stability of the unheated and heat-induced EWPs at pH 7.0. A–D: unheated EWP, 2.5% IMO-EWP, 10% IMO-EWP and 50% IMO-EWP, respectively; E–H: heat-induced EWP, 2.5% IMO-EWP, 10% IMO-EWP and 50% IMO-EWP, respectively. Different letters (a–d) on the column indicate significant differences (*p* < 0.05).

Proteins can be adsorbed at the oil-water interface to form a coherent viscoelastic layer to stabilize the oil. As shown in Figure 8, EAI and ESI of the conjugates (B–D) and their nanoparticles (F–H) were remarkably higher than EWP (A). Moreover, the EAI and ESI of the nanoparticles from conjugates were increased about 3-folds and 2-folds, respectively, as compared with EWP. This can be explained by the increased *H*_o_ induced by MR and heat treatment [5] which could promote the adsorption of protein on oil-water interface and shielded the oil droplets of emulsions against aggregation [31]. This agrees with previous reports that the emulsifying properties of EWP or its constituent proteins were improved through MR or heat treatment [13,32]. In addition, the ESI of conjugates and their nanoparticles were enhanced with the increase of DG, which was likely related to the increased electrostatic repulsion (Figure 4). In conclusion, the heat-induced nanoparticles formed from EWP conjugates showed the best emulsifying activity and stability.

## 4. Conclusions

Glycation of EWP with IMO changed the conformation of EWP and improved its thermal stability and surface hydrophobicity. Moreover, the increased absolute values of ζ-potential after glycation would enhance the electrostatic repulsion among the IMO-EWP molecules when pH was above p*I*. As a result, thermal aggregation of IMO-EWP conjugates was significantly suppressed and transparent nanoparticle dispersions (with particle diameter <200 nm) were obtained after heating at pH 7.0–9.0. This demonstrated that the heat-induced nanoparticles showed the highest emulsifying activity and stability. The present study made a successful attempt in fabrication of heat-induced nanoparticles with improved emulsifying activity and stability at mild pH and salt conditions. It provided the supplement for EWP nanoparticle application on neutral conditions, compared to the aggregates fabricated at extremely acid and alkaline conditions. Further investigation on the storage stability and encapsulation of bioactive compounds using nanoparticles will expand its application in many food systems, such as EWP beverages or stabilizers for emulsions.

## Figures and Tables

**Table 1:** DSC assessment of denaturation temperature (*T*_d_) and the associated enthalpy change (Δ*H*) of EWP and IMO-EWP conjugates at pH 7.0.

Samples	Peak 1		Peak 2	
	*T*_d_ (°C)	Δ*H* (J/g)	*T*_d_ (°C)	Δ*H* (J/g)
EWP	66.56 ± 0.04	0.67 ± 0.03	84.11 ± 0.05 ^a^	3.94 ± 0.02 ^a^
2.5% IMO-EWP	-	-	84.06 ± 0.12 ^a^	3.07 ± 0.04 ^b^
10% IMO-EWP	-	-	87.19 ± 0.02 ^b^	2.84 ± 0.05 ^c^
50% IMO-EWP	-	-	89.17 ± 0.06 ^c^	2.74 ± 0.02 ^c^

Different letters (a–c) in the same column indicate significant differences (*p* < 0.05).

## Supplementary Materials

The following are available online at http://www.mdpi.com/2079-4991/8/11/943/s1, Figure S1: High performance liquid chromatography (HPLC) of IMO, Table S1: Composition of IMO determined by HPLC (Figure S1).
