# Supplementary material for: Application of Glycation in Regulating the Heat-Induced Nanoparticles of Egg White Protein
Source: Nanomaterials (Basel). 2018 Nov 15;8(11):943. doi: 10.3390/nano8110943 (PMC6266673; doi:10.3390/nano8110943)
Supplement: Supplementary file 1 [file nanomaterials-08-00943-s001.pdf]

Supplementary Materials

Application of glycation in regulating the heat-induced nanoparticles of egg white protein

Chenying Wang [1], Xidong Ren [2,3], Yujie Su [1]*and Yanjun Yang [1]*

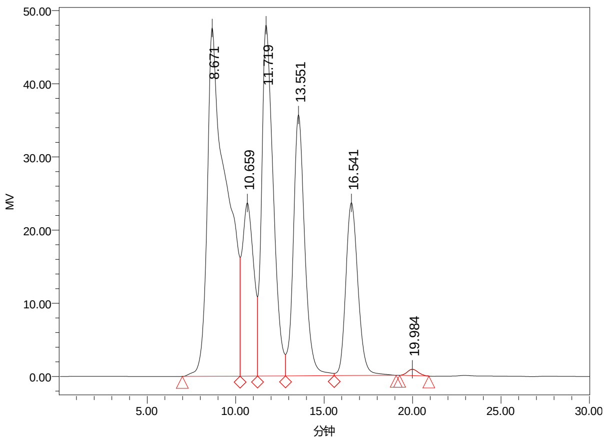

Figure S1. High performance liquid chromatography (HPLC) of IMO

Table S1. Composition of IMO determined by HPLC (Figure S1)

Name	Retention time (min)	Peak area	Relative peak area (%)
＞tetrasccharides	8.671	3585928	37.21
tetrasccharides	10.659	1082286	11.23
trisaccharides	11.719	2186799	22.69
disaccharides	13.551	1614226	16.75
monosaccharides	16.541	1130189	11.73
	19.984	36876	0.38
